# Transcriptional analysis of cleft palate in TGFβ3 mutant mice

**DOI:** 10.1038/s41598-020-71636-0

**Published:** 2020-09-10

**Authors:** J. Liu, S. K. Chanumolu, K. M. White, M. Albahrani, H. H. Otu, A. Nawshad

**Affiliations:** 1 Department of Oral Biology, College of Dentistry, University of Nebraska Medical Center, Lincoln, NE 68583 USA; 2 Department of Electrical and Computer Engineering, University of Nebraska-Lincoln, Lincoln, NE 68588 USA; 3 Department of Growth and Development, College of Dentistry, University of Nebraska Medical Center College of Dentistry, Lincoln, NE 68583 USA

**Keywords:** Computational biology and bioinformatics, Developmental biology

## Abstract

Cleft palate (CP) is one of the most common craniofacial birth defects, impacting about 1 in 800 births in the USA. Tgf-β3 plays a critical role in regulating murine palate development, and *Tgf-β3* null mutants develop cleft palate with 100% penetrance. In this study, we compared global palatal transcriptomes of wild type (WT) and *Tgf-β3* −/− homozygous (HM) mouse embryos at the crucial palatogenesis stages of E14.5, and E16.5, using RNA-seq data. We found 1,809 and 2,127 differentially expressed genes at E16.5 vs. E14.5 in the WT and HM groups, respectively (adjusted *p* < 0.05; |fold change|> 2.0). We focused on the genes that were uniquely up/downregulated in WT or HM at E16.5 vs. E14.5 to identify genes associated with CP. Systems biology analysis relating to cell behaviors and function of WT and HM specific genes identified functional non-Smad pathways and preference of apoptosis to epithelial-mesenchymal transition. We identified 24 HM specific and 11 WT specific genes that are CP-related and/or involved in Tgf-β3 signaling. We validated the expression of 29 of the 35 genes using qRT-PCR and the trend of mRNA expression is similar to that of RNA-seq data . Our results enrich our understanding of genes associated with CP that are directly or indirectly regulated via TGF-β.

## Introduction

Orofacial clefting is the most common craniofacial anomaly treated in pediatric hospitals and is the second most common birth defect, with a prevalence ranging from 1/500 to 1/2,500 in humans^1,2^. Formation of a confluent palate is a precise orchestration of many palatal cellular processes, including cellular movement, cell death, and cell cycle progression^3,4^. Many genes have been implicated in the etiology of cleft palate (CP). Transforming growth factor-beta (*Tgf-β*) isoforms (1, 2, and 3) are essential for proper development, including palate fusion^5,6^. The roles of *TGF-β* in palatogenesis, participating in different phases of palate development, such as elevation, contact, and fusion, have been detailed previously^3^. A number of studies suggest that *TGF-β3* is a candidate gene for causing cleft palate in mice^2^, chickens^7^, and humans^8^. The *Tgf-β3* homozygous knockout (stated as homozygous, HM in this study) mouse model presents the phenotype of CP but no other major anomalies^9^.

**Figure 1:**
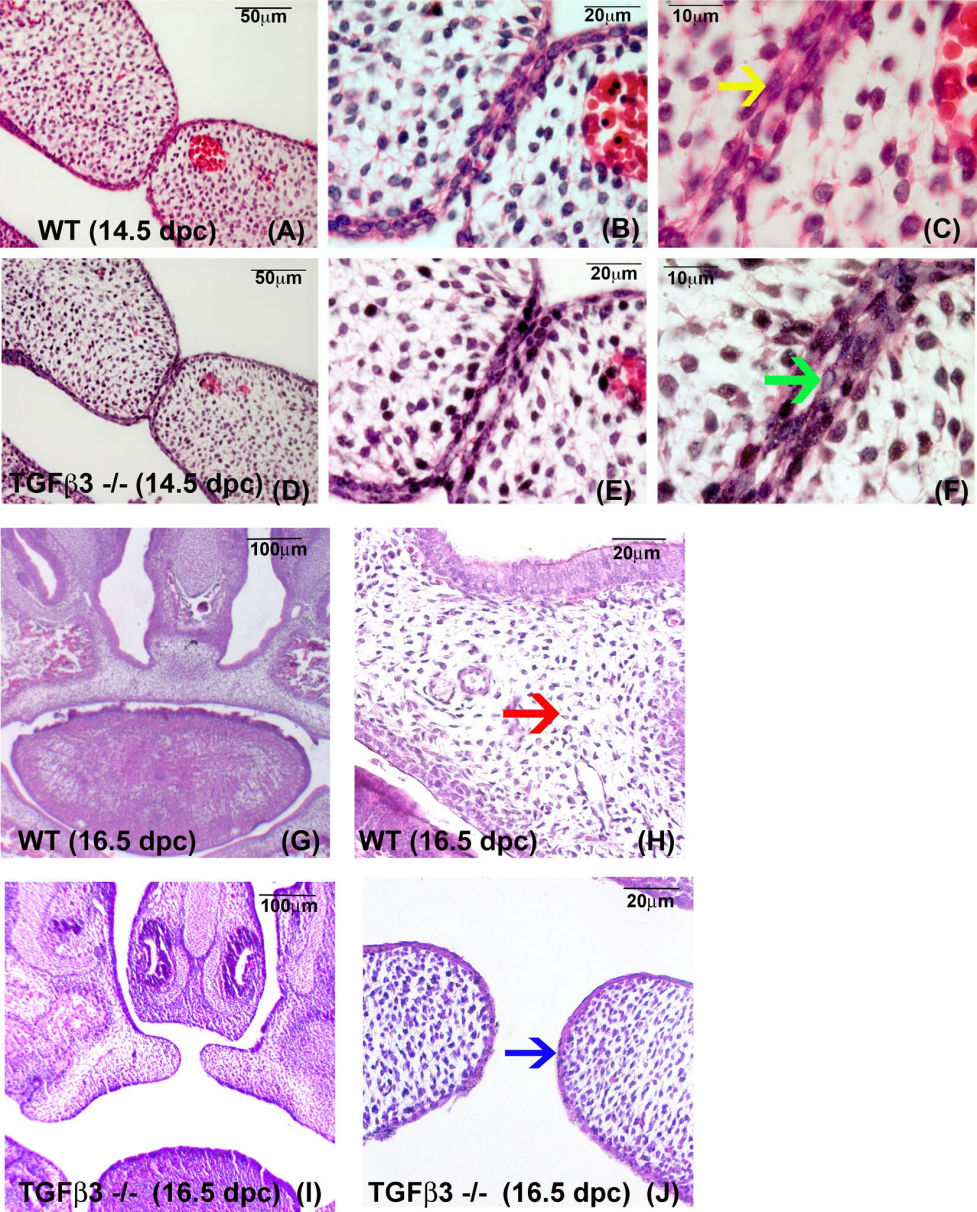
Histological illustration of murine palate formation in WT (**A**–**C**, **G**, **H**) and TGFβ3 −/− (HM) mice (**D**–**F**, **I**, **J**). At 14.5 dpc, in WT palates the mid-palate region shows a complete union of a two-cell thick, basal medial edge epithelium (MEE), consisting of two opposite palates forming a tight epithelial seam in low (**A**) and higher magnifications (**B**); (**C**, yellow arrow). At 14.5 dpc, the HM (TGFβ3 −/−; **D**–**F**) palates demonstrate a trapped additional layer of flattened epithelium between the MEE of opposite palatal shelves (**D**); (**E**); (**F**, green arrow) [Figs. A–F are from J. Cell. Physiol. 230: 1,212–1,225, 2015. Wiley Periodicals, Inc., copyright John Wiley & Sons, Inc.]. At 16.5 dpc, the WT palates show complete disintegration of the seam and palatal mesenchymal confluence (**G**), higher magnification (**H**, red arrow). At 16.5 dpc, TGFβ3 −/− (HM) palates fail to fuse (**I**), higher magnification (**J**), and drift apart as face continues to grow, resulting in cleft palate consequently. In the HM palates at 16.5 dpc, the palatal epithelia are stratified (**J**, blue arrow).

The process of palatogenesis is remarkably similar among vertebrates. In mice, the palatal shelves *grow* out bilaterally from the internal surfaces of the maxillary processes (~ E11.5). The shelves first vertically *elongate* on either side of the tongue (~ E12.5) and then *elevate*, becoming horizontal above the tongue as the tongue descends (~ E13.5). When the opposing shelves approach each other, the cells of the outer layer (periderm) covering the medial edge epithelia (MEE) slough off (~ E14,0), exposing the lateral surfaces of the underlying basal MEE cells to close contact with each other (also known as *“adherence”* or *“fusion”)*, promoting formation of the *midline epithelial seam (MES)* (~ E14.5; Fig. 1A–C). The palatal epithelial seam subsequently disintegrates and reaches complete palatal mesenchymal confluency (by ~ E16.5; Fig. 1G,H). This process is governed by epithelial mesenchymal transition (EMT) and/or apoptosis^4^, resulting in the mesenchymal portion of the two palatal shelves becoming continuous.

While both the wild type (WT) and HM palates proceed through the exact same stages of palatogenesis until E14.0 (i.e., growth, elongation, and elevation), the persistence of periderm on the *Tgf-β3* HM palates at the final “adherence/fusion” stage at E14.5 hinders palatal MEE contact between opposite palatal shelves (~ E14.5, Fig. 1D–F) resulting in palatal cleft (Fig. 1I, J). It is evident that the MEE contact is strictly regulated by *Tgf-β3* in WT to ensure desquamation of the periderm for immaculate fusion^10,11^. The absence of *Tgf-β3* fails to facilitate desquamation of the periderm and hinders fusion resulting in subsequent palatal cleft in HM. Therefore, in this study, we aim to assess the difference in the temporal gene expression for the WT and *Tgf-β3* HM samples from E14.5 to E16.5 to provide further understanding of genes functionally regulated by *TGF-β3* during murine palatal fusion and examine their contribution to the development of CP using our previously established RNA-seq data^12^.

Several studies have explored CP-related genes using RNA-seq in murine and human CP samples^13–15^, however, downstream molecular mechanisms directly and indirectly controlled by *Tgf-β3* signaling remain largely unexplored. We previously used RNA-seq to obtain the transcriptome of WT and *Tgf-β3* HM mice at crucial stages of palatogenesis (E14.5 and E16.5)^12^. However, our previous study was confined to only assessing the expression profile of the 322 known CP genes in our RNA-seq dataset and did not explore the genes differentially expressed between the WT and HM groups and/or between the E14.5 and E16.5 time points. In this study, using the same RNA-seq data established in our previous paper, we applied new and advanced data quality control, filtering, quantification, normalization, differential expression, and systems biology approaches that were not previously employed, and explored all of the differentially expressed genes across all of the genotypes and time points. This way, we could identify potential key molecular components and possible underlying mechanisms of palate formation during development, failure of which may result in CP.

In particular, we identified the differentially expressed genes between genotypes (at a given time point) as well as the differentially expressed genes that changed temporally from E14.5 to E16.5 (for a given genotype). We further applied a comparative temporal transcriptome analysis of *Tgf-β3* WT and HM mice and identified genes that were uniquely regulated by each genotype, which may contribute to CP formation in HM mice. Our systems biology analysis based on the Gene Ontology (GO) and Ingenuity Pathway Analysis (IPA; QIAGEN Inc., https://www.qiagenbioinformatics.com/products/ingenuity-pathway-analysis) platforms identified the biological functions, molecular networks, and regulatory pathways (especially in relation to TGF-β signaling) for the differentially expressed genes. Finally, we validated the RNA-seq analysis results with real-time quantitative polymerase chain reaction (qRT-PCR) for 29 genes that are either known CP-related genes and/or genes that are regulated by TGF-β and fall under its downstream signaling pathways. For all of the 29 genes, the degree and direction of differential expression based on RNA-seq analysis agreed with the qRT-PCR results. Collectively, this data will enrich our understanding of TGF-β signaling during palate development and provide insight into the temporal regulation of downstream TGF-β-regulated genetic modulators that control cell morphology, cellular differentiation, apoptosis, and morphogenesis of the embryo.

## Results

Our previously acquired RNA-seq data^12^ involving wild type and *Tgf-β3* homozygous knockout mouse models on E14.5 and E16.5 represented four sample groups (denoted as “WT14.5,” “WT16.5,” “HM14.5,” and “HM16.5”), each represented by two biological replicates (denoted as “a,” or “b”) for a total of eight samples. The average read count for the raw RNA-seq data was ~ 65.5 M paired-end reads (i.e., ~ 130.1 M total reads) per sample providing a high coverage of the transcriptome. The re-analysis of this data involved data trimming and filtering, normalization, expression quantification, clustering, differential analysis, comparative analysis, systems biology analysis, and qRT-PCR validation.

### RNA-seq data processing and clustering

After trimming and filtering, the number of average total reads per sample came down to ~ 128.1 M (Supplementary Fig. 1A). The average read length was 101 bp in the raw data, which decreased to 95.86 bp following trimming and filtering (Supplementary Fig. 1B). On the other hand, the average Phred read quality score increased to 36.79 from 35.84 (Supplementary Fig. 1C); and the percentage of high-quality bases (bases with a quality score > 20) per sample increased to 99.20% from 96.28% (Supplementary Fig. 1D) following trimming and filtering. Therefore, both the total number of reads and the average read length parameters showed small changes in quantity after trimming and filtering; but this resulted in significant data quality improvement.

RNA-seq analysis generated expression data for 103,215 transcripts. Transcripts that showed a TPM value of less than 1 in both samples in all of the four groups were eliminated from downstream analysis leaving 52,475 transcripts. Biological replicates showed high degree of correlation (r > 0.98). In Supplementary Fig. 2, we show the hierarchical clustering of the samples using all 52,475 transcripts. This global unsupervised grouping reveals that the samples were separated clearly by time as there are two main clades where one clade only consists of E14.5 samples and the other clade only consists of E16.5 samples. The effect of genotype on the transcriptional profiling was subtle because in the clustering tree we do not see a grouping based on the genotype neither globally, nor within a time point. Hence, there is a need for supervised analysis methods to identify the differences in gene expression due to genotypic variance. Furthermore, the height of the branching points in Supplementary Fig. 2 both for the E14.5 and E16.5 sample groups implied that the similarity between the WT and HM groups was higher at E14.5 than it was at E16.5. This is because of the shorter branch lengths among samples in the E14.5 clade compared to the branch lengths among samples in the E16.5 clade. Therefore, the samples at E16.5 showed a more divergent transcriptional profile and thus the effects of *Tgf-β3* knockout were more pronounced at E16.5.

**Figure 2:**
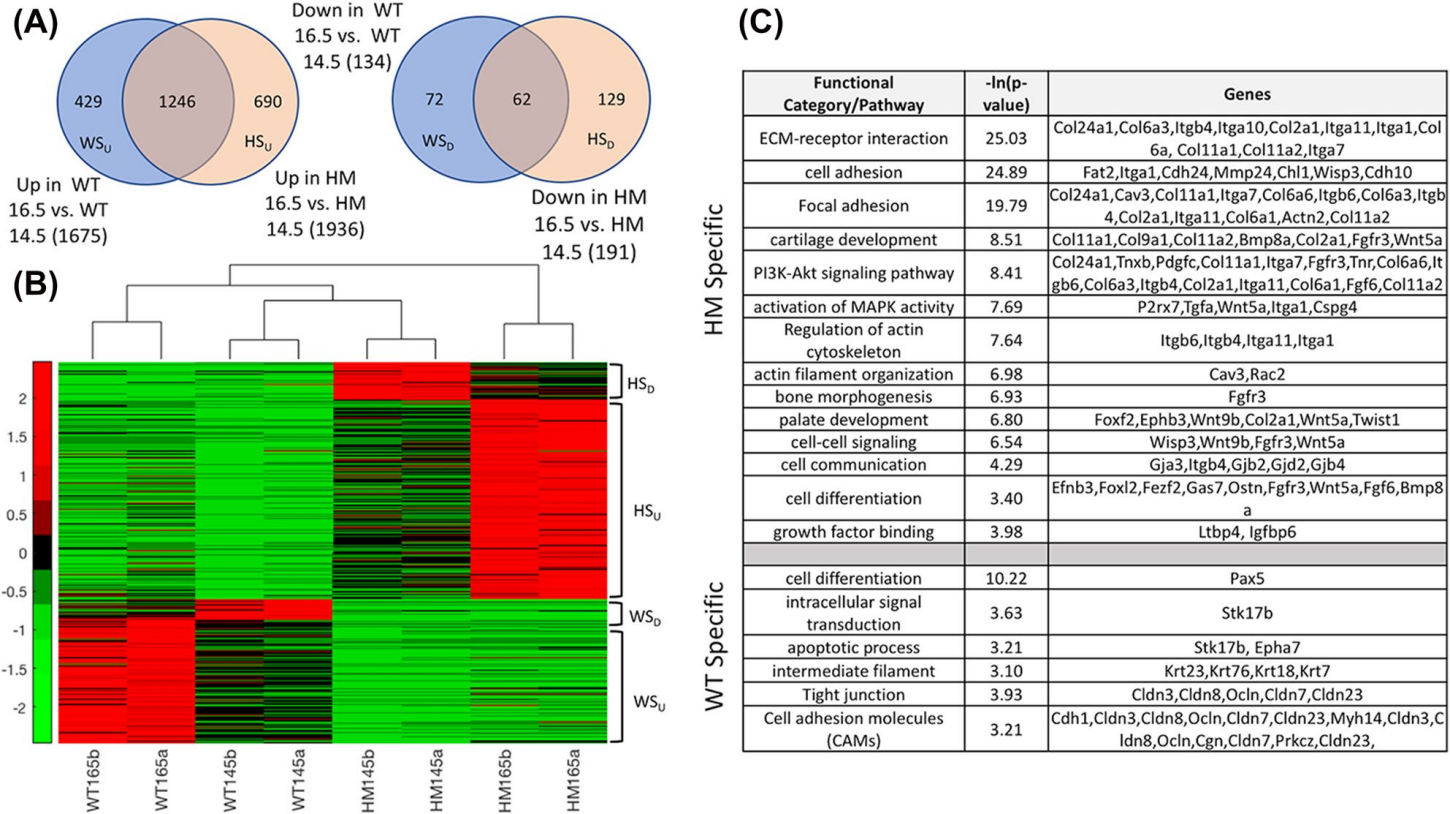
Hierarchical clustering of 501 (429 + 72) WT-specific and 819 (690 + 129) HM specific genes with the color bar showing the row z-score. (**A**) Comparison of significantly differentially expressed genes (SDEGs) between E16.5 and E14.5 in the WT and HM groups. WT-Specific Up (WS_U_): genes uniquely upregulated in the WT group at E16.5; WT-Specific Down (WS_D_): genes uniquely downregulated in the WT group at E16.5; HM-Specific Up (HM_U_): genes uniquely upregulated in the HM group at E16.5; HM-Specific Down (HM_D_): genes uniquely downregulated in the HM group at E16.5, (**B**) hierarchical clustering of all four groups (eight samples) using the 501 (429 + 72) WT-specific and 819 (690 + 129) HM-specific genes identified in (**A**), (**C**) Significantly enriched Gene Ontology (GO) categories in the WT-specific and HM-specific gene lists (sample genes in the groups are shown), fold change of relevant WT- and HM-specific genes.

### Differential expression analysis

Differential expression analysis also showed the stark temporal difference in gene expression between samples (Table 1). There were 4,115 and 5,304 significantly differentially expressed genes (SDEG) between the E16.5 and E14.5 time points for the WT and HM samples, respectively indicating that the temporal transcriptional change in HM samples was more widespread than in normal controls. Furthermore, in both genotypes, the number of upregulated genes with time (2,421 for WT and 3,153 for HM) was more than the number of downregulated genes (1,694 for WT and 2,151 for HM) showing that transcriptional induction overshadowed transcriptional silencing. This difference in up/downregulation was further emphasized when we applied a 2.0-fold change (FC) cutoff (on top of the adjusted *p* < 0.05 cutoff) to define SDEGs. Most of the upregulated genes survived this FC cutoff (1,675 out of 2,421 in WT and 1,936 out of 3,153 in HM had an |FC|> 2.0); but the number of SDEG that were downregulated decreased dramatically (134 out of 1,694 in WT and 191 out of 2,151 in HM had an |FC|> 2.0). This further underlined the trend of significant transcriptional induction with time.

**Table 1:** Significantly differentially expressed genes (SDEG), multiple hypothesis testing corrected *p* value < 0.05, across time and genotype points listed separately for up/downregulation (FC, fold change).

Comparison	Group	Gene list	No. of SDEG	No. of SDEG (|FC|> 2.0)
Between time points	WT	Up in WT E16.5 versus WT E14.5	2,421	1,675
		Down in WT E16.5 versus WT E14.5	1694	134
	HM	Up in HM E16.5 versus HM E14.5	3,153	1,936
		Down in HM E16.5 versus HM E14.5	2,151	191
Between genotype	E14.5	Up in HM E14.5 versus WT E14.5	8	0
		Down in HM E14.5 versus WT E14.5	5	0
	E16.5	Up in HM E16.5 versus WT E16.5	30	10
		Down in HM E16.5 versus WT E16.5	8	3

We observed a very subtle difference between the two genotypes at a given time point. At days E14.5 and E16.5, there were only 13 and 38 SDEGs between the WT and HM samples, respectively. In concordance with the unsupervised hierarchical clustering results, we saw a greater difference between the genotypes at E16.5. This was further strengthened by observing 13 SDEGs between the two genotypes with an |FC|> 2.0 at E16.5, while there were no SDEGs between the WT and HM samples with an |FC|> 2.0 at E14.5.

### WT specific and HM specific gene identification

Since a direct comparison between the HM and WT groups yielded a very subtle difference, we defined the effect due to *Tgf-β3* knockout by comparing the temporal SDEGs in the two groups. Following our adjusted *p* < 0.05 and |FC|> 2.0 cutoffs, we compared the 1,675 and 1,936 SDEGs that were upregulated in E16.5 vs. E14.5 in the WT and HM groups, respectively. We identified 429 genes that were upregulated in the WT group, but not in the HM group; similarly, we identified 690 genes that were upregulated in the HM group, but not in the WT group. Conversely, we compared the 134 SDEGs downregulated in E16.5 vs. E14.5 in the WT group with the 191 SDEGs downregulated in E16.5 vs. E14.5 in the HM group. We identified 72 genes that were downregulated in the WT group, but not in the HM group and similarly we identified 129 genes that were downregulated in the HM group, but not in the WT group. These results are summarized in Fig. 2A. We called the 501 (429 + 72) SDEGs uniquely up/downregulated in the WT group “WT specific”; and similarly, we called the 819 (690 + 129) SDEGs uniquely up/downregulated in the HM group “HM specific.”

In Fig. 2B, we show the hierarchical clustering of the 1,320 (501 + 819) WT and HM specific SDEGs across the four sample groups. This visual representation of expression values (heatmap) clearly shows how WT specific genes are significantly up/downregulated between WT E14.5 and WT E16.5 samples, whereas the expression of these genes remains “not significantly altered” between the HM E14.5 and HM E16.5 samples. A similar (but complementary) observation holds for the HM-specific genes. Figure 2B also shows the sample similarity based on genes specific to the genotypes, which clustered the biological replicates together and grouped the samples in E14.5 under the same clade. This implies that the temporal difference in the dataset was more dominant than the genotypic difference as we do not see samples from the same genotype in the two different time points group together. The HM samples at E16.5 stood out as a distinct group, which is reasonable as the phenotypic difference between the genotypes was not visible at E14.5 but emerged clearly at E16.5, making the HM samples at E16.5 a distinctly separate phenotypic group among the four.

### Functional analysis

In order to understand the genes and corresponding signaling network and functional mechanisms that would explain the differences observed between HM and WT samples across E16.5 and E14.5 time points, we highlighted the GO functional categories and KEGG pathways that were statistically significantly enriched in the WT and HM specific gene lists (Fig. 2C). A complete list of SDEGs, enriched GO categories and KEGG pathways can be found in the Supplementary Data. In Table 2, we list 24 HM-specific and 11 WT-specific genes based on their functional relevance by any one or more of the following criteria: (1) falling under the TGF-β signaling pathway, (2) contributing to palatogenesis or cleft palate in mice, (3) being involved in palatal cellular processes, such as EMT, apoptosis, differentiation, proliferation that are functional in palatal and craniofacial morphogenesis, and (4) being directly or indirectly regulated by TGF-β signaling.

**Table 2:** Selected genes that are uniquely significantly differentially expressed (adjusted *p* value < 0.05) in the HM or WT groups.

ENSEMBLE gene ID	Gene name	Adjusted *p* value	Fold change (HM E16.5/HM E14.5)	Function
**HM specific**				
24,330	Col11a2*	1.72E−27	6.16	Variants involved in CP^58,59^
27,966	Col11a1*	2.63E−24	3.71	Variants involved in CP^58,59^
32,060	Cryab*	5.97E−10	3.44	EMT regulator^60,61^ Apoptosis inhibitor^62,63^
54,555	Adam12*	2.26E−19	2.9	TGFβ-induced EMT regulator^64^ TGFβ signaling modulator^65^ Epithelial cell proliferation and apoptosis regulator^66^
22,483	Col2a1*	3.50E−06	2.68	Mutations involved in cleft palate^58,59^
31,391	L1cam*	1.59E−04	2.38	Apoptosis resistance^67^ EMT regulator^68^ Cell adhesion^69^
26,253	Chrng*	1.40E−02	2.21	Mutations associated with cleft palate^70^
5,148	*Klf5*	1.17E−03	2.19	Epithelial proliferation promotor^71^ Apoptosis inhibition^72^
22,037	Clu*	4.54E−05	2.18	TGFβ signaling modulator^73^ Apoptosis inhibitor^74^ TGFβ-induced EMT regulator^75^
24,529	Lox*	3.14E−06	2.15	EMT regulator^76,77^ TGFβ signaling target^78^
20,598	Nr-cam*	8.95E−03	2.15	Cell proliferation and motility stimulator^79^
24,778	Fas*	4.34E−03	2.1	EMT inducer^80^ Apoptosis mediator^81,82^
28,763	Hspg2*	2.78E−04	2.03	TGFβ signaling target^83^
22,512	Cldn1*	1.32E−03	2.01	EMT promotor^84,85^ Apoptotic regulator^86,87^
20,758	*Itgb4*	4.08E−03	2	EMT promotor^88^ Epithelial Cells mobility enhancer^89,90^ Apoptosis inhibitor^91^
35,799	Twist1*	2.85E−13	− 2.17	CLP candidate gene^92,93^ EMT marker^94–96^ TGFβ signaling regulator^96,97^
28,019	*Pdgfc*	1.74E−09	− 2.21	CP candidate gene^1,98,99^ TGFβ signaling target^100^
21,994	Wnt5a*	4.44E−04	− 2.48	TGFβ signaling crosstalk^101–103^ EMT mediator^104,105^
2,930	*Ppp1r17*	2.98E−02	− 2.52	Embryonic palate development regulator^25^
48,450	*Msx1*	6.95E−10	− 2.66	CP candidate gene^106–108^ EMT mediator^109^ TGF-β superfamily pathways regulator^106^
40,310	*Alx4*	2.16E−13	− 2.67	CP candidate gene^110^ EMT mediator^111,112^
59,022	*Kcp*	7.83E−10	− 2.67	TGF-β superfamily pathways regulator^113^
37,034	*Pax1*	3.22E−03	− 2.77	Embryogenesis regulator^114^ Apoptosis and differentiation inducer^115–117^
18,486	*Wnt9b*	3.59E−05	− 4.88	CLP candidate gene^118,119^ Facial outgrowth and fusion promoter^120^ Cell proliferation, differentiation and cell polarity regulator^121,122^

ENSEMBLE gene ID	Gene name	Adjusted *p* value	Fold change (WT E16.5/WT E14.5)	Function
**WT specific**				
21,638	Ocln*	5.78E−10	2.84	Tight junction component^123^ Adhesion, apoptosis, differentiation and homeostasis regulator in keratinocytes^124^
27,858	Tspan2*	2.86E−07	2.49	Cell adhesion, proliferation, differentiation and migration regulator^26^ TGF-β signaling downstream effector^26^
21,678	F2rl1*	1.73E−03	2.26	TGF-β signaling target^125,126^ Cell proliferation enhancer^127,128^
42,228	Lyn*	1.37E−07	2.17	EMT mediator^129^ Apoptosis inhibitor^130^ TGF-β signaling target^131^
303	Cdh1*	5.24E−03	2.09	CLP candidate gene^5,132,133^
63,727	*Tnfrsf11b*	3.11E−03	2.04	TGF-β signaling pathways mediator^134,135^
21,614	Vcan*	4.85E−30	− 2.66	Cell proliferation, adhesion and apoptosis regulator^136,137^ Apoptosis inhibitor^138,139^
28,487	*Bnc2*	3.05E−05	− 2.76	Embryonic craniofacial mesenchymal cell multiplication regulator^140^
30,498	*Gas2*	8.17E−05	− 3.06	Cell cycle and apoptosis regulator^141^ TGF-β signaling pathways target^142^
41,911	Dlx1*	7.45E−09	− 3.25	Craniofacial patterning controller^143^ TGF-β signaling pathways inhibitor^144,145^
33,487	*Fndc3c1*	2.04E−21	− 4.58	Ectoderm differentiation gene^146^

CP, Cleft Palate; CL, Cleft Lip; CLP, Cleft Lip and Palate; identified in mouse or in human (*).

### Systems biology analysis

**Figure 3:**
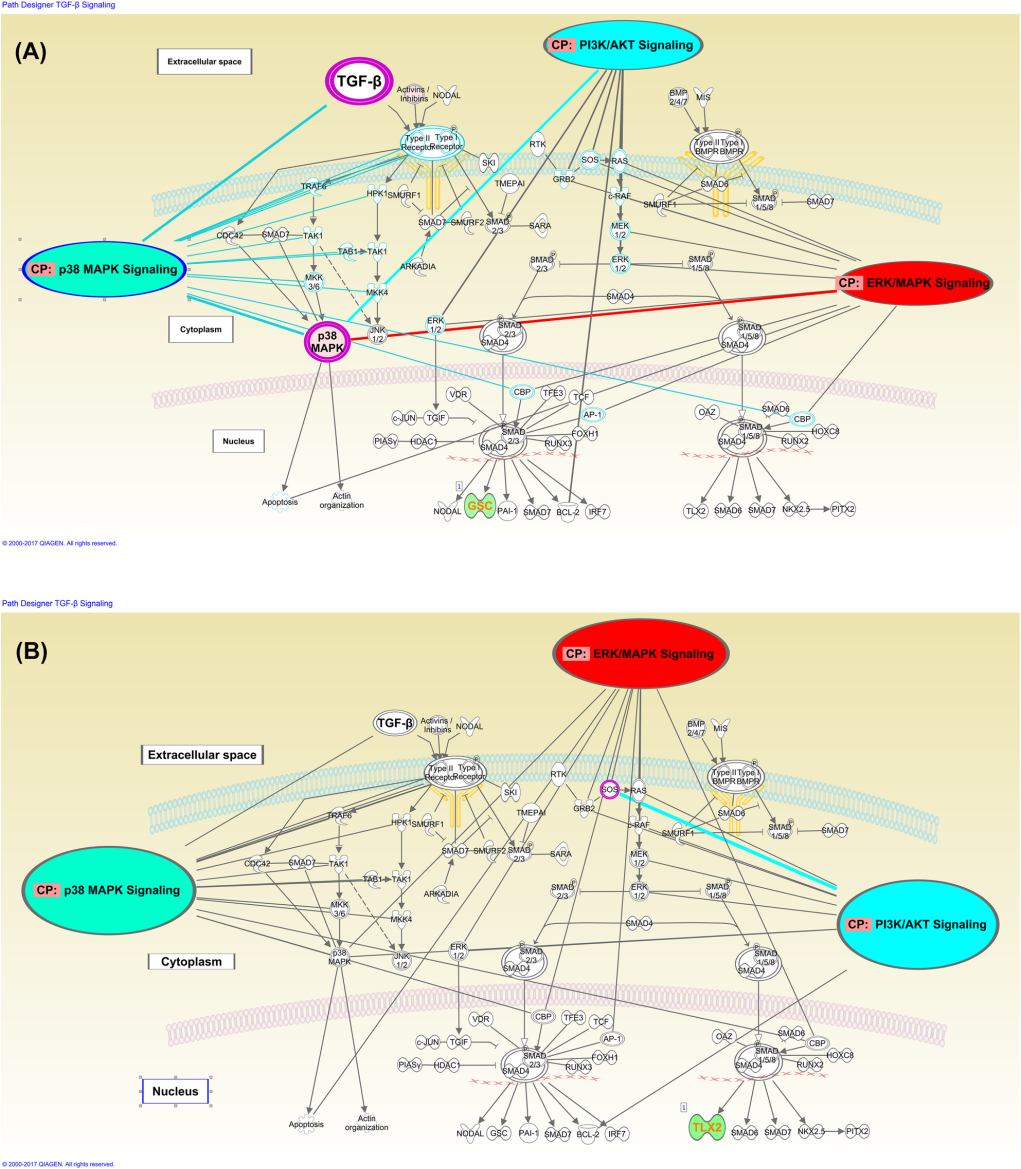
(**A**) IPA TGF-β signaling canonical pathway analysis overlaying the WT specific gene list: genes significantly differentially expressed (adjusted *p* < 0.05) with a |FC|> 2.0 uniquely in WT, E16.5 versus E14.5. Pink implies upregulation and green implies downregulation. Genes that are also involved in major non-Smad pathways (ERK/MAPK, p38MAPK and PI3-AKT) in palatogenesis are indicated with links to the corresponding pathways. (**B**) IPA TGF-β signaling canonical pathway analysis for the HM specific gene list: genes significantly differentially expressed (adjusted *p* < 0.05) with a |FC|> 2.0 uniquely in HM, E16.5 versus E14.5. Green implies downregulation. Genes that are also involved in major non-Smad pathways (ERK/MAPK, p38MAPK and PI3-AKT) in palatogenesis are indicated with links to the corresponding pathways.

Using IPA, we analyzed the signaling networks and crosstalks amongst the downstream molecules that could be uniquely functional in WT and HM palates. Our network analysis results suggest that the TGF-β, ERK/MAPK, p38MAPK and PI3K/AKT pathways directly regulate *p38MAPK* in WT (Fig. 3A and Supplementary Fig. 3), which is not the case in HM (Fig. 3B and Supplementary Fig. 7). The canonical pathway analysis using IPA further strengthened this observation when we specifically considered the TGF-β signaling pathway separately for WT specific genes (Fig. 3A) and for HM specific genes (Fig. 3B). Furthermore, we showed that *Goosecoid* (*Gsc*) is downregulated by TGF-β signaling in WT (Fig. 3A), whereas, in the HM, TGF-β signaling downregulates transcription factor *Tlx2* (Fig. 3B). Finally, our results suggest that, in both in WT and HM, TGF-β signaling regulates expression of neither Smads nor of transcripts encoding proteins that act upstream or downstream of Smads (Fig. 3A,B; Supplementary Figs. 3 and 7).

We identified WT specific genes that are also TGF-β downstream target genes. When we overlaid the Epithelial Mesenchymal Transition (EMT) functional category on these genes, we observed that only two genes were selected (Supplementary Fig. 4a). When we similarly overlaid the “cell death” functional category on the same gene list, we observed nine selected genes (Supplementary Fig. 5a). These results potentially demonstrate that in WT, TGF-β signals to regulate “cell morphology” and “differentiation” that facilitate apoptosis over EMT as the number of TGF-β downstream target genes are predominantly pro-apoptotic genes but not EMT. We further analyzed the mechanistic networks generated by IPA that involve TGF-β signaling. These networks show the interaction among upstream regulators that best explain the changes observed in the SDEG list. We performed this analysis for WT specific genes and found that these upstream regulators were predominantly pro apoptotic, (12 out of 14, Supplementary Fig. 5b) not EMT (none out of 14, Supplementary Fig. 4b) genes.

**Figure 4:**
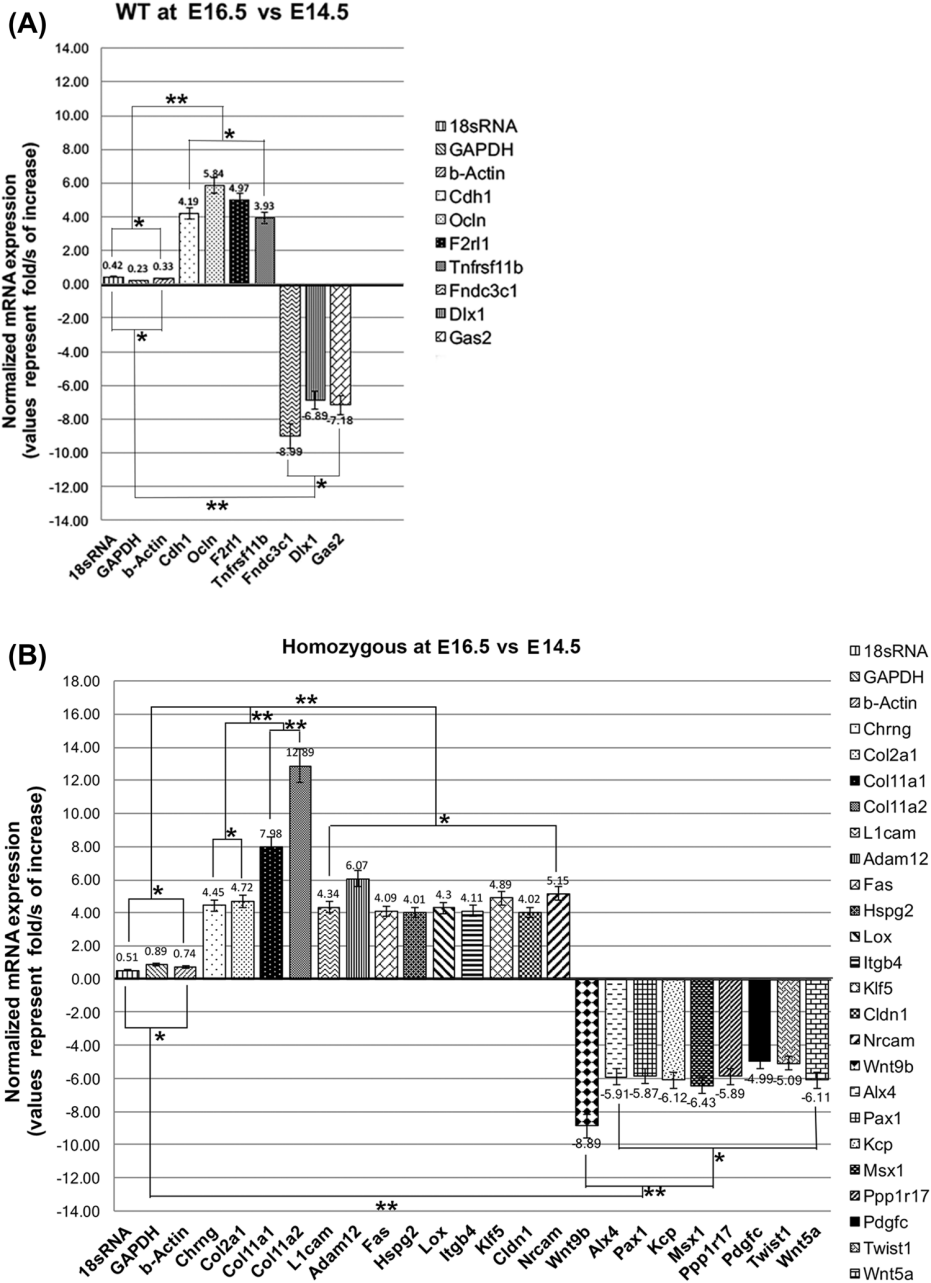
(**A**) Illustration of the level of mRNA expression showing fold change (log2) expression of the WT gene mRNA comparative to reference control genes (18rSRNA, GAPDH, and β-actin). The bar heights demonstrate mean expression of the genes in the WT samples. And the error bars suggest 95% confidence interval estimates of the mean expressions. One asterisk designates statistically significant difference between the means of a sample set in comparison to the mean of the control sample set (*p* value < 0.05); two asterisks indicate statistically significant difference (*p* value < 0.01). (**B**) Illustration of the level of mRNA expression showing fold change (log2) expression of HM gene mRNA comparative to reference control genes (18rSRNA, GAPDH, and β-actin). The bar heights demonstrate mean expression of the genes in HM samples. And the error bars suggest 95% confidence interval estimates of the mean expressions. One asterisk designates statistically significant difference between the means of a sample set in comparison to the mean of the control sample set (*p* value < 0.05); two asterisks indicate statistically significant difference (*p* value < 0.01).

### qRT-PCR validation

We further assessed the expression levels of the 29/35 differentially expressed genes listed in Table 2 using real-time qRT-PCR. Our qRT-PCR results were all in agreement with our RNA-seq results (Table 2), which demonstrated that in WT, *Cdh1*, *Ocln*, *F2rl1,* and *Tnfrsf11b* were uniquely upregulated (folds of increase: 4.19, 5.84, 4.97, and 3.39, respectively) while *Fndc3c1*, *Dlx1,* and *Gas2* were uniquely downregulated (folds of increase: − 8.99, − 6.89, and − 7.18, respectively) at E16.5 when compared to E14.5 (Fig. 4A). Similarly, in HM palates, genes that uniquely showed upregulation at E16.5 in RNA-seq data (Table 2), including *Chrng*, *Col2a1*, *Col11a1*, *Col11a2*, *L1cam*, *Adam12*, *Fas*, *Hspg2*, *Lox*, *Itgb4*, *Klf5*, *Cldn1,* and *Nrcam*, exhibited significantly increased mRNA expression in qRT-PCR (folds of increase > 4.02), when compared to E14.5 (Fig. 4B). Similarly, qRT-PCR results verified that *Wnt9b*, *Alx4*, *Pax1*, *Kcp*, *Msx1*, *Ppp1r17*, *Twist1,* and *Wnt5a* were also in concordance with RNA-seq data, presenting downregulated mRNA expression (folds of decrease <  − 4.99) uniquely in HM (Fig. 4B).

## Discussion

All isoforms of TGF-β are involved in both palatal epithelial and mesenchymal cell proliferation, differentiation, transformation, and apoptosis^16^. TGF-β3 plays a distinctive role in periderm removal, palatal fusion and seam disintegration chronologically that involves crosstalk and reciprocal signaling between the two cell populations by epithelial-mesenchymal interaction (EMI)^17,18^, which cannot be achieved by other isoforms^10,11^. Therefore, in this study, we analyzed *Tgf-β3* HM whole palates to accurately explain the global TGF-β3 signaling during palate development. Usage of both epithelial and mesenchymal cells is a standard practice as previously done^19,20^. In one of the most elegant studies of the palatal transcriptome analysis by NIH Facebase consortium, the authors also used regions of palate with a combination of both palatal epithelia and mesenchyme^13^.

Our differential expression analysis showed a very subtle difference between the WT and HM palates—there were only 13 and 38 SDEGs between the WT and HM samples, respectively, at days E14.5 and E16.5 (see Supplementary Data). In agreement with the unsupervised hierarchical clustering data, we noticed a greater difference between the genotypes at E16.5. This difference was not only in numbers but also in the degree of differential expression. When the |FC|> 2.0 cutoff was employed, there were no SDEGs at E14.5 and only 13 SDEGs at E16.5 between the HM and WT groups (Table 1). This observation may be attributable to the obvious phenotypical difference, a palatal cleft at E16.5 and no difference at E14.5. Interestingly, 9 out of the 13 SDEGs in HM vs. WT at E16.5 were epithelial-specific genes with a majority of them being involved in palate deformities and cleft palate (Table 3). Henceforth, the differences between these two genotypes mainly impacted palatal epithelial cell-specific function, which is in accordance with the expression of TGF-β3 in WT or lack of it in HM*.*

**Table 3:** Epithelial-specific genes that are significantly differentially expressed (adjusted *p* value < 0.05, |FC|> 2.0) at E16.5 in HM versus WT.

ENSEMBLE gene ID	Gene name	Adjusted *p* value	Fold change (HM E16.5/WT E16.5)	Function
26,908	Eif2s3y*	4.39E−76	16.75	Mutations associated with CLP^147^
20,592	Kdm5d*	2.35E−95	14.13	Mutations associated with palate deformities^148^
22,290	Uty*	4.81E−45	8.69	Mutations associated with palate deformities^149^
26,900	Ddx3y*	1.77E−25	5.78	Mutations associated with cleft palate^150^ TGF-β signaling pathways target^151^
20,248	*Serpinb3a*	0.000523	2.15	Epithelial hyperplasia and hyperkeratosis^152,153^
245,026	Col6a6*	0.001059	2.13	Epithelial hyperkeratosis^154^
16,682	*Krt4*	0.000106	2.09	Epithelial integrity gene^155^ Epithelial cell differentiation modulator^156^
333,564	*Fndc3c1*	0.000915	2.02	Ectoderm differentiation gene^146^
57,294	Rps27*	0.000126	− 2.75	Mutations associated with cleft palate^157^

CLP, Cleft Lip and Palate; identified in mouse or in human (*).

The subtle difference in gene expression at a given time point is likely to trigger larger downstream changes through direct and/or indirect regulatory signaling that results in a phenotypic difference. Such a large effect would be observed through variations in the temporal gene expression patterns for the two genotypes, which also accounts for any subtle cell population differences as direct comparisons between the genotypes are not involved. Therefore, we focused on genes that show temporal changes in WT (from E14.5 to E16.5) due to the presence of *Tgf-β3*, which are not identified as changed in HM (from E14.5 to E16.5) due to the absence of *Tgf-β3* and vice versa. In order to identify genes that show temporal difference uniquely in the WT or HM groups, we identified WT specific and HM specific gene lists (Fig. 2A). The functional analysis of these gene lists (Fig. 2C, Supplementary Data) render GO categories and KEGG pathways that are statistically significantly enriched in the WT or HM, such as the “apoptotic process,” “cell adhesion molecules (CAMs),” and “focal adhesion.” These lists show specific signaling pathways and TGF-β downstream molecules modulating both the epithelial and the mesenchymal cellular functions that are unique to WT and HM, resulting in immaculate palatogenesis in WT but cleft in HM.

In the Supplementary Data, we also list the 1,308 genes commonly up/down regulated between E16.5 and E14.5 both in WT and HM groups. The rationale behind the table is to describe the common genes that are responsible for phases that are common in both WT and HM, such as palatal cell proliferation, differentiation, transformation and apoptosis. These cellular changes are ongoing in both genotypes, hence, the genes listed in this Supplementary Data represent a common palatal cellular behavior and function that may not necessarily imply genes that can trigger cleft palate. Of note among commonly upregulated genes are *Sntn*, which participates in cell growth/maintenance and is involved in cell communication process in the nasopharynx^21^, and *Tmem212*, which codes for a transmembrane protein known to interact with transcription factor *Tcf12*, an important paralog of *Tcf4*^22^ and *Forkhead box k2 (Frk2)*. *Frk2* is an important regulator palatal cell proliferation via activation of the phosphoinositide 3-kinase/AKT pathway^23^. Other genes to names are, *Ppp1r32*, which is a substrate for cGMP-dependent protein kinase and is involved in central nervous system development and intracellular signal transduction^24^, is upregulated in both WT and HM palates. It implements protein serine/threonine phosphatase inhibitor activity and inhibits phosphatase activities of *protein phosphatase 1 (Pp1)* and protein *phosphatase 2A (Pp2A)* complexes. Pp1 and Pp2A were reported by Weston et al.^25^ to account for virtually all detectable serine/threonine protein phosphatase activity during the development of embryonic palate. We also observed common upregulation of *Tspan1* and *33* in both WT and HM going from E14.5 to E16.5. *Tspan1*have been shown to play crucial roles in biologic processes including cell adhesion, proliferation, differentiation, and migration^26^. Our data suggest that the role of *Tspans* may be limited to cellular proliferation and differentiation via Smad pathways. Another commonly upregulated transcript is *Tnfrsf11a* (*RANK*), which is a key regulator of bone homeostasis^27^. As early as E14.5, mesenchymal condensations undergo chondrogenesis initially and ultimately membranous ossification that give rise to the hard palate^28^. We expect the palatal cells express RANK to regulate osteoclast function in palatal bone formation in both genotypes. *In terms of epithelial markers, Krt13*, which has been shown to be expressed in the suprabasal layer of stratified palatal keratinized epithelia^29^, is also among the commonly upregulated transcripts. *Krt4*, a type II cytokeratin, is specifically expressed in differentiated layers of all of oral mucosal epithelia along with family member *Krt13*^30^. Similar to *Krt13*, *Krt4* is also upregulated in both WT and HM. Showing a similar expression pattern to *Krt13* among commonly upregulated transcripts is *Sprr3* (aka Loricrin), a marker for terminally differentiated keratinized and non-keratinized oral mucosa^31^. This is potentially due to the fact that palatal epithelia undergo significant stratification with terminally differentiated keratinized epithelia covering the oral side of the palatal lining in both WT and HM palates.

Using IPA, we identified several novel findings which may suggest, and potentially imply, new signaling network molecules as well as cellular functions. Our data suggests that TGF-β signaling may induce palatogenesis through regulating *p38MAPK* in WT palates (Fig. 3A,B, Supplementary Figs.3 and 7). These findings are in agreement with previous work^32–34^ showing *p38MAPK* activation by TGF-β signaling during palatogenesis. It has been previously demonstrated that in palatogenesis TGF-β3 signals through the SMAD pathway^35–38^. Our results indicate no change in SMADs and its down- and up-stream genes at the mRNA levels both for WT and HM palates (Fig. 3A,B, Supplementary Figs. 3 and 7). This observation does not necessarily imply an inactivation of the SMAD pathway as transcriptional level activity does not always imply protein level functionality. On the other hand, these results may imply *non-Smad* pathways to be also at play both in the WT and HM while in HM, unlike WT, this signaling cascade does not include *p38MAPK* (Supplementary Figs. 3 and 7).

Uniquely in the WT palate*, Goosecoid* (*Gsc*), a homeobox-containing gene, is downregulated by *TGF-β* signaling in palatogenesis (Fig. 3A). *Gsc* mutant mice display defects in the pharyngeal muscles and the pharyngeal mucosa^39^. *TGF-β* signaling is known to promote EMT by regulating the *Gsc* gene during embryonic Spemann’s organizer formation^40^ as well as breast cancer metastasis^41^. Since GSC is known to be a homeobox transcription factor that promotes EMT, it is likely that apoptosis is favored for seam disintegration since an EMT gene, such as *Gsc* is downregulated in WT palates. Similarly, uniquely in the HM, *Tgf-β* signaling downregulates a separate homeobox gene, *Tlx2*, a transcription factor (Fig. 3B). The *Tlx2* gene encodes transcription factors essential in the development of neural-crest-derived cells suggesting a physiological role in the transcription-factor cascade underlying the differentiation of neuronal lineages during embryogenesis^42^. We did not find any direct relationship between TGF-β and *Tlx2* but based on the fact that *Tlx2* is crucial for neural-crest-derived cell development, it is therefore likely that palatal epithelia (which is an ectoderm derived cell) has limited or no role for *Tlx2* and therefore it is downregulated.

Our upstream regulator analysis identified targets of *Tgf-β* in the WT specific gene list. Exploring the involvement of these genes in the EMT and cell death mechanisms revealed limited involvement (*Cdh1* and *Lef1*) of the EMT pathway (Supplementary Fig. 4a) whereas nine apoptotic genes (*Ace, Cdh1, Dcn, Dlx2, Krt18, Pparg, Rasgrp, Sema7a, and Tnfrsf11b)* were directly regulated by *TGF-β* (Supplementary Fig. 5a). We also identified mechanistic networks, which are interaction networks of upstream regulators that explain the changes observed in the WT (or HM) specific genes. When we explored the functional characteristics of these interconnected upstream regulators, none of the genes were involved in the EMT (Supplementary Fig. 4b); and 12 out of 14 genes were involved in “cell death” (Supplementary Fig. 5b).

We identified genes regulated by *Tgf-β3* in WT specific genes and showed that among these *Tgf-β3* targets, *Cdh1* is upregulated and *Lef1* is downregulated (Supplementary Fig. 6). Loss of *Cdh1* is a key marker of EMT^43^; and the expression of *Cdh1* can be repressed by the transcription factor *Lef1* in palatal EMT^22,44^. Additionally, anti-EMT cell–cell adhesion genes (*Cdh1*, *Ocln*, *Tspan2*) are upregulated, which may suggest a potential relationship between *Tgf-β3* signaling and suppression of palatal EMT. These findings may indicate two possible outcomes: (a) since palatal seam disintegration is complete at E16.5, the EMT markers are no longer expressed, or (b) EMT may not be a mechanism of palatal seam disintegration. Our findings, based on mechanistic network and upstream regulator analysis (Supplementary Figs. 4a, 4b, 4a, 4b, and 4), suggest that the latter is more probable as the number of apoptotic regulatory network genes are significantly more than the number of EMT genes.

## Conclusion

Identifying transcripts that play key roles in regulating palatal development in critical stages has been a powerful approach to understanding how *Tgf-β3* controls normal palatogenesis and how the lack of signaling (and its downstream signaling partners) is associated in induction of cleft palate. This study identifies potential CP-related genes based on differential expression between genotypes and gestational ages. The data presented in this work provide a strengthened understanding of the complex genetic mechanism of *Tgf-β3-*regulated palatogenesis. In addition, we discussed the variations in gene expression in the absence of *Tgf-β3* in HM implicated in cleft palate. Our results represent a comprehensive analysis of the gene profile in murine cleft palate due to the absence of *Tgf-β3*. Further elucidation of the significantly up/downregulated genes will enhance our understanding of the mechanisms controlling palate development, thereby paving the way for prevention of cleft palate during development.

## Methods

### Animal selection and breeding

*Tgf-β3* heterozygous (+/−) C57BL/6J breeder mice were obtained from Tom Doetschman (BIO5 Institute, University of Arizona, AZ). The reproduction and genotyping of *Tgf-β3 *−/− mice was conducted as previously described^9^. Mice were accommodated and subject to procedures at the University of Nebraska Medical Center (UNMC) College of Dentistry Animal Facilities under the approval of the UNMC Institution Animal Care and Use Committee (IACUC # 06–064). Null mutant embryos were generated by intercrossing *Tgf-β3* heterozygous male and female mice in a Mendelian fashion.

### Genomic DNA purification and genotyping

Palatal tissues were dissected under the NIKON SMZ1000 stereo microscope system (NIKON, Tokyo, Japan) from embryos collected on embryonic day (E) 14.5 and E16.5 following the identification of vaginal plugs, which are considered to be E0.5. Palatal samples were stored in RNA*later* Stabilization Reagent (QIAGEN, Hilgen, Germany) to preserve the gene expression profile and individually labeled and matched with the corresponding tail tissue used for genotyping as detailed in our previous study^12^.

### Extraction of RNA, construction of small RNA libraries, and RNA-Seq

Two biological replicates from each genotype and gestational stage were designed to ensure reproducibility and rule out the possibility of differences caused by technical procedures. Palatal shelves were harvested in pairs from eight fetuses out of four litters. Each sample consisted of two pairs of palatal shelves dissected from fetuses of the same genotype from the same litter. The total RNA was purified using Arcturus PicoPure RNA Isolation Kit (THERMOFISHER SCIENTIFIC, San Francisco, CA) to reliably extract high-quality RNA from a few cells by following the manufacturer’s protocols. Purity and concentration were measured by ultraviolet spectroscopy (NANODROP, Wilmington, DE). The RNA integrity evaluation, construction of libraries, and validation was performed as described in our previous study^12^.

### RNA-seq analysis

RNA-seq data was obtained for four groups: two genotypes, *Tgf-β3* −/− HM and *Tgf-β3* +/+ WT samples, profiled at two time points, embryonic days E14.5 and E16.5. Each group was represented by two biological replicates, resulting in eight samples. Each sample was run in two lanes on the ILLUMINA HISEQ2000 next generation sequencer using the 2 × 101 bp paired-end mode. Due to the high correlation coefficient between them (r > 0.995) and in order to increase the coverage per biological sample and reduce the lane effect^45^, lane data for each sample were pooled at the read level.

Raw reads were analyzed with FASTQC (V. 0.11.5) for quality control^46^. Overrepresented (e.g., adapter and similar technical) sequences remaining in the raw reads were assessed and subsequently removed using TRIMMOMATIC (V. 0.36) in the palindrome mode based on default alignment detection and scoring parameters^47^. Trimmomatic was also used for low quality base filtering. Maximum information quality filtering was employed with a minimum average read quality threshold of 25. Following technical sequence and low-quality base removal, reads that were shorter than 36 bp were filtered out.

Transcript quantification was done based on the GRCm38.p5 reference genome using Salmon (v. 0.8.2) with default parameters^48^. Salmon uses sample-specific models, such as correction for GC-content bias, that improve the accuracy of transcription abundance estimates. We used transcripts per million (TPM) in Salmon’s output as the normalized relative abundance measure employed in our downstream analysis. Differential gene expression analysis was done using DESeq2 (Love, Huber et al. 2014), which has been shown to perform well in experimental designs with few replicates^49^. The RNA-seq data used in this paper were deposited under NCBI’s Gene Expression Omnibus (GEO) database (Accession No.: GSE109838).

Clustering of samples and/or genes was done using the Unweighted Pair Group Method with Arithmetic Mean (UPGMA) method (also known as hierarchical clustering) with Pearson’s correlation as the distance measure^50^. The expression data matrix was row-normalized prior to the application of average linkage clustering. The Database for Annotation, Visualization and Integrated Discovery (DAVID) v6.7^51^ was used for functional analysis of the gene lists, interrogating the Biological Process (BP), Molecular Function (MF), and Cellular Component (CC) Gene Ontology (GO) categories^52^, and the Kyoto Encyclopedia of Genes and Genomes (KEGG) pathways^53^. Biologically relevant categories that are overrepresented in the gene set and, therefore, may be of further interest were assessed using the Expression Analysis Systematic Explorer (EASE) score in the DAVID tool. The EASE score is the upper bound of the distribution of jackknife iterative resampling of Fisher’s exact probabilities with Bonferroni multiple testing correction. Categories containing low numbers of genes are underweighted so that the EASE score is more robust than the Fisher exact test. The EASE score is a significance level, with smaller EASE scores indicating increasing confidence in overrepresentation. We selected GO categories that have EASE scores of 0.05 or lower as significantly overrepresented.

We further analyzed the differentially expressed gene lists using the Ingenuity Pathway Analysis (IPA; QIAGEN Inc., https://www.qiagenbioinformatics.com/products/ingenuity-pathway-analysis) software. IPA is based on the manual curation of scientific literature to identify pathways, networks, and functional categories that are significantly represented in the input gene list^54^. The computational analysis methods used in IPA are based on enrichment approaches^55,56^ where pathways or functional groups in which the input gene lists are overrepresented are identified. By the same token, IPA identifies upstream regulators (e.g., transcription factors, microRNAs, kinases, compounds, or drugs) and generates interaction networks (based on known interactions identified in the literature) that best explain the transcriptional changes observed in the input gene list.

### Confirmation of differentially expressed genes with qRT-PCR

To verify differentially expressed genes in *Tgf-β3* WT and HM samples, qRT-PCR was undertake as previously described^19,57^. Embryonic palates were extracted from at E14.5 and E16.5, and RNA extraction was conducted as previously described^19,57^ using Arcturus PicoPure RNA Isolation Kit (THERMOFISHER SCIENTIFIC, San Francisco, CA) to reliably extract high-quality RNA from a few cells. RNA (500 ng) was converted to cDNA using Invitrogen Superscript IV VILO Master Mix (THERMOFISHER SCIENTIFIC, San Francisco, CA) that generated a significant cDNA yield at high temperatures in less time. An additional preamplification step was performed using TaqMan PreAmp Master Mix (THERMOFISHER SCIENTIFIC, San Francisco, CA) with a custom preamplification pool of genes of interest to amplify small amounts of cDNA without introducing amplification bias. Samples were preamplified for 14 cycles with thermal cycling conditions of 95 °C for 15 s and 60 °C for 4 min followed by immediate placement on ice. Finally, samples were diluted with TE buffer (ph 8.0) to 1:20 and were placed on 96-well custom array plates in technical triplicate and qRT-PCR executed with TaqMan Fast Advanced Master Mix (THERMOFISHER SCIENTIFIC, San Francisco, CA) reagents. Polymerase chain reaction conditions were run in 40 cycles at 95 °C for 15 s, and 60 °C for 60 s. Following subtraction of technical repeat’s gene-specific Δ^CtCt^ value from the housekeeping gene’s Δ^CtCt^ value, the data were, then, analyzed using analysis of variance (ANOVA) on the repeat-normalized ΔΔ^CtCt^ values, including the control group; and translate effects were estimated from the ANOVA onto the multiplicative scale. The values of 29 genes tested were normalized by adjusting for the concentration of established housekeeping gene, like 18S rRNA, glyceraldehyde 3-phosphate dehydrogenase (GAPDH) and β-actin and the Δ^Ct^ values of a naive/vehicle group.

### Ethics approval and consent to participate

*Tgf-β3* heterozygous (+/−) C57BL/6 J breeder mice were obtained from Tom Doetschman (BIO5 Institute, University of Arizona, AZ). The reproduction and genotyping of *Tgf-β3* −/− mice was conducted as previously described^9^. The live mice experiments were performed in accordance with the guidelines and regulations of the University of Nebraska Medical Center (UNMC) College of Dentistry Animal Facilities under the approval of the UNMC Institution Animal Care and Use Committee (IACUC # 18-088).

## Supplementary information

Supplementary file1.

Supplementary Datasets.

## Data Availability

The RNA-seq data used in this paper were deposited under NCBI’s Gene Expression Omnibus (GEO) database (Accession No.: GSE109838).
