# Supplementary material for: Transcriptional analysis of cleft palate in TGFβ3 mutant mice
Source: Sci Rep. 2020 Sep 10;10:14940. doi: 10.1038/s41598-020-71636-0 (PMC7483747; doi:10.1038/s41598-020-71636-0)
Supplement: Supplementary file 1 — Supplementary file1. [file 41598_2020_71636_MOESM1_ESM.docx]

**Supplementary Material**

**Tile:**

Transcriptional analysis of cleft palate in TGFβ3 mutant mice

**Authors:**

Liu J^*√^, Chanumolu SK^#√^, White KM^^^, Albahrani M^#^, Otu HH^#^ and Nawshad A^°*^

**Authors Affiliations:**

^*^ Department of Oral Biology, College of Dentistry, University of Nebraska Medical Center, Lincoln NE-68583

^^^ Department of Growth and Development, College of Dentistry, University of Nebraska Medical Center College of Dentistry, Lincoln, NE-68583.

^#^ Department of Electrical and Computer Engineering, University of Nebraska-Lincoln, Lincoln, NE-68588.

^√^ Co-first authors

**^°^Corresponding author:**

Ali Nawshad, PhD

Associate Professor

Department of Oral Biology,

College of Dentistry

University of Nebraska Medical Center,

4000 E Campus loop

Lincoln, NE- 68583

Tel: 402-472-1378

Fax: 402-471-2551

Email: [anawshad@unmc.edu](mailto:anawshad@unmc.edu)

**Supplementary Figure 1.** **(A)** Total number of paired-end reads (in logarithmic scale), **(B)** average read length in base pairs (bp), **(C)** average read quality, and **(D)** % of bp in the ensemble of reads that exceed a quality score of 20.


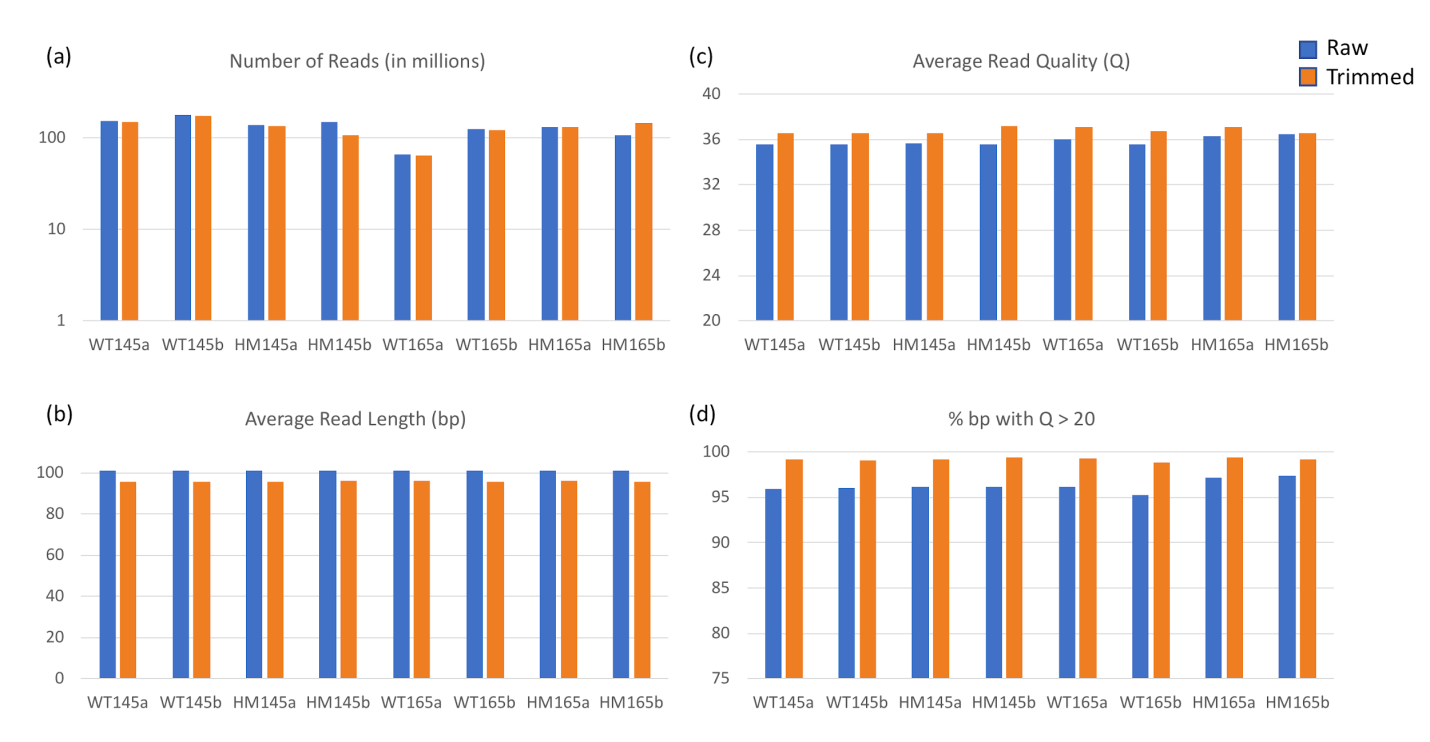


**Supplementary Figure 2:** Hierarchical clustering of all of the samples using all 52,475 transcripts measured with TPM>1 in at least one sample group.


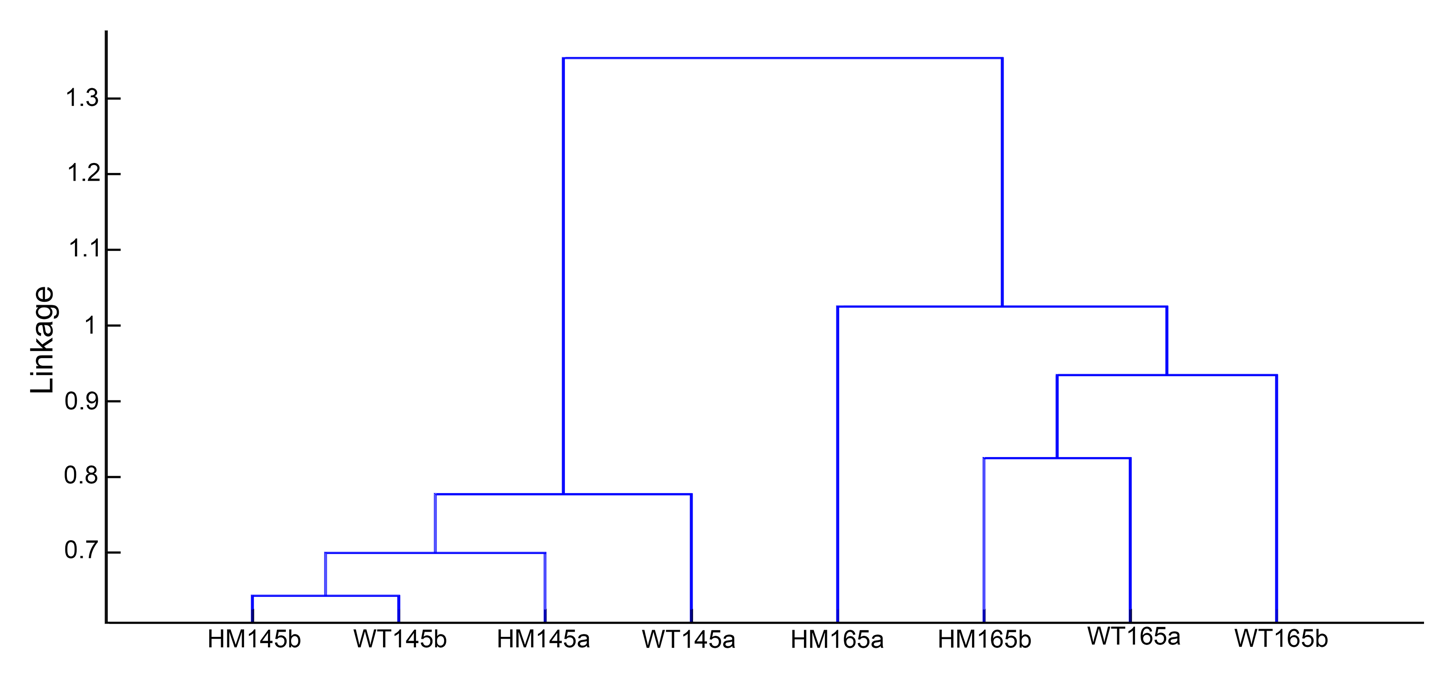


**Supplementary Figure 3.** IPA Interaction Network Analysis for the WT specific gene list: genes significantly differentially expressed (adjusted p<0.05) with a |FC| > 2.0 uniquely in WT, E16.5 vs. E14.5. Genes are color coded such that pink implies upregulation and green implies downregulation. Genes that are also involved in major non-Smad pathways (ERK/MAPK, p38MAPK and PI3-AKT) in palatogenesis are indicated with links to the corresponding pathways. The p38MAPK gene appears as a central hub node that has been upregulated in E16.5. A solid line implies a direct interaction while a dashed line implies an indirect interaction


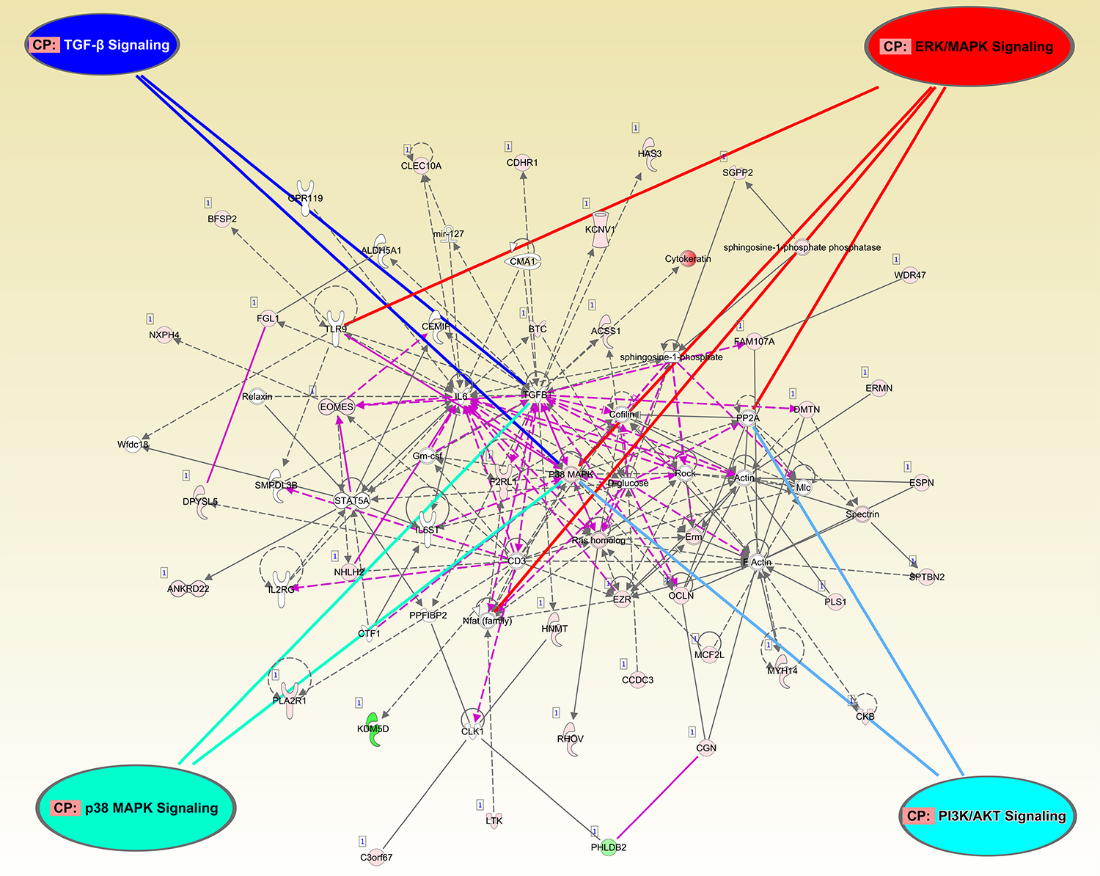


**Supplementary Figure 4a**

**a.** IPA upstream regulator analysis of TGF-β 1 and 3 for the WT specific gene list: genes significantly differentially expressed (adjusted p<0.05) with a |FC| > 2.0 uniquely in WT, E16.5 vs. E14.5. Shades of red implies upregulation and shades of green implies downregulation. Arrows highlight the WT specific genes regulated by TGF-β 1 and/or 3. Genes that are involved in Epithelial-Mesenchymal Transition (EMT) are highlighted. A solid line implies a direct interaction while a dashed line implies an indirect interaction


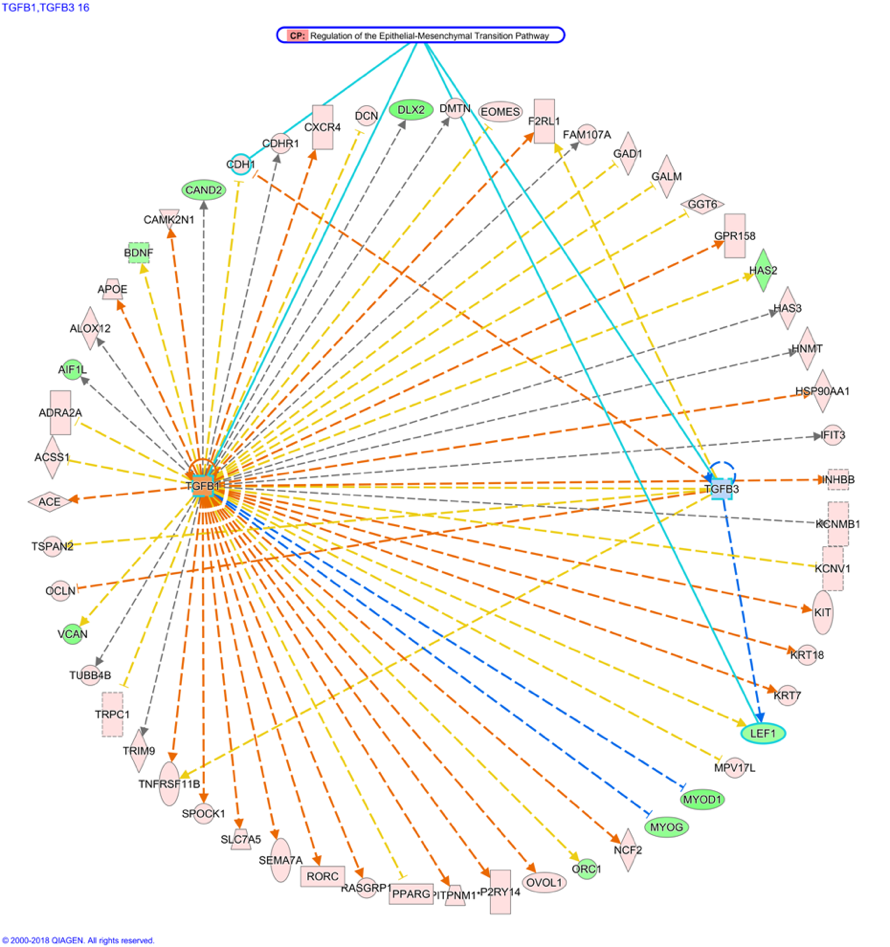


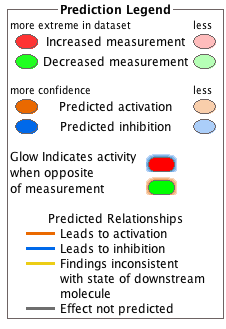


**Supplementary Figure 4b**

**b.** IPA mechanistic network depicting the interaction between upstream regulators that explain the observed changes in the WT specific gene list: genes significantly differentially expressed (adjusted p<0.05) with a |FC| > 2.0 uniquely in WT, E16.5 vs. E14.5. Regulators that are involved in Epithelial-Mesenchymal Transition (EMT) are highlighted (none observed, no purple border). A solid line implies a direct interaction while a dashed line implies an indirect interaction


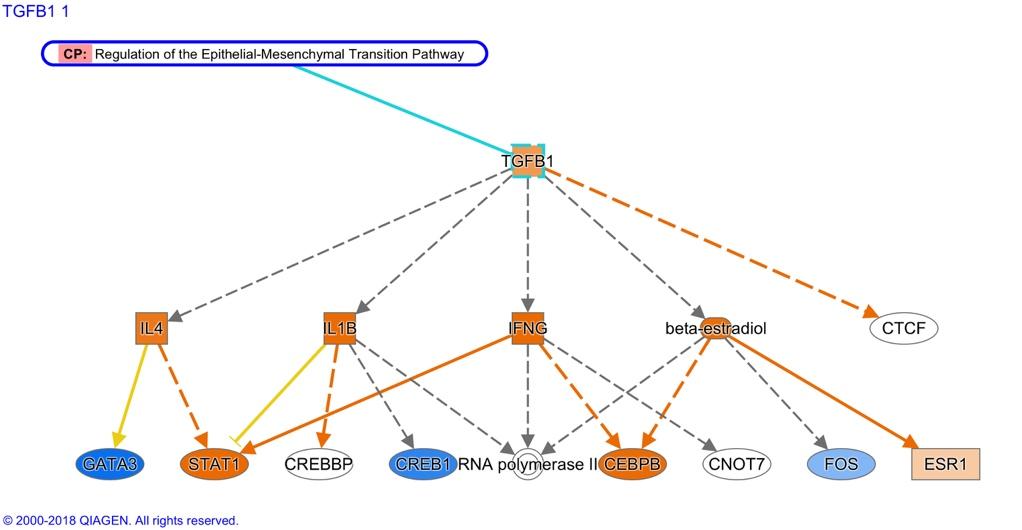


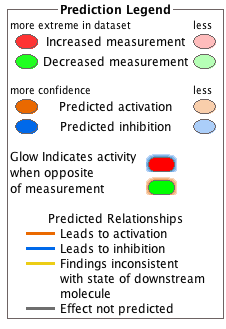


**Supplementary Figure 5a**

**a.** IPA upstream regulator analysis of TGF-β 1 and 3 for the WT specific gene list: genes significantly differentially expressed (adjusted p<0.05) with a |FC| > 2.0 uniquely in WT, E16.5 vs. E14.5. Shades of red implies upregulation and shades of green implies downregulation. Arrows highlight the WT specific genes regulated by TGF-β 1 and/or 3. Genes that are involved in the “cell death” functional category are highlighted (in purple border). A solid line implies a direct interaction while a dashed line implies an indirect interaction


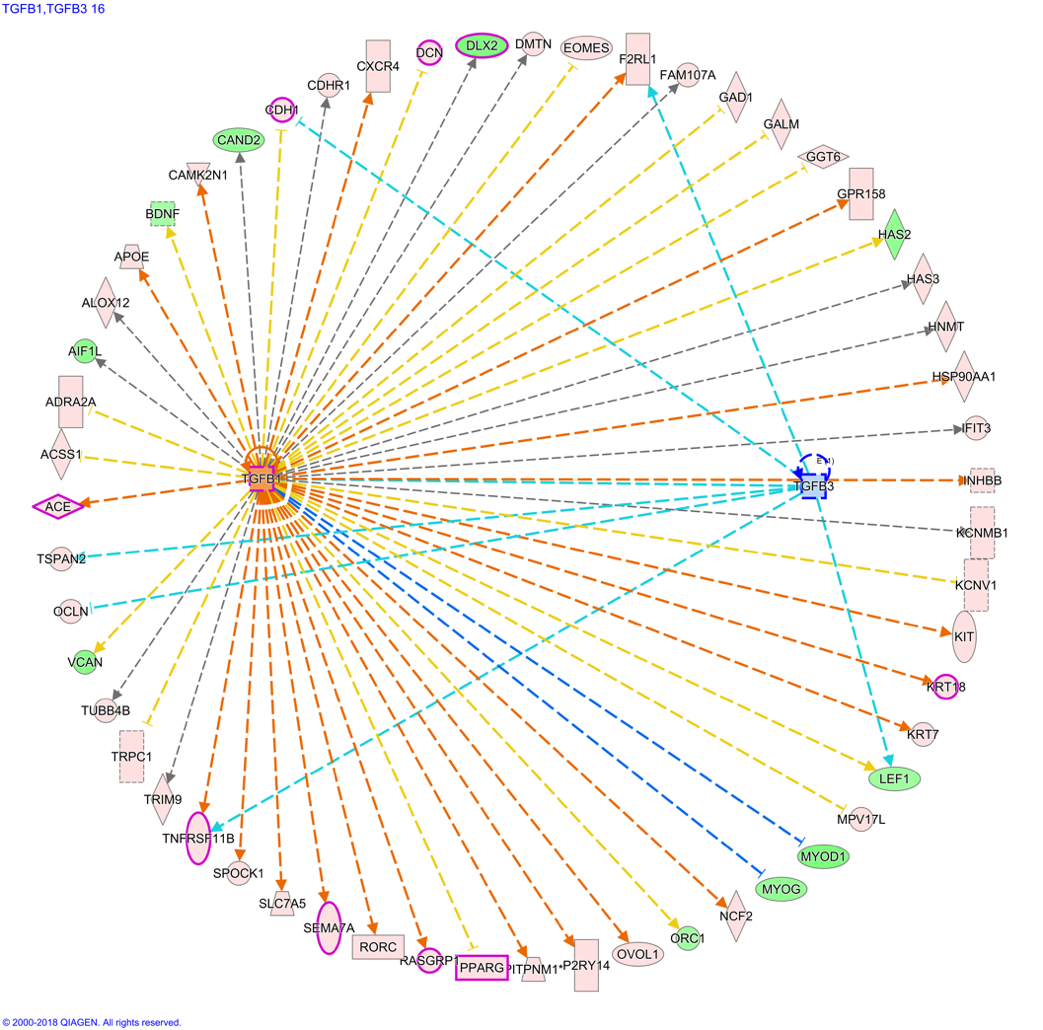

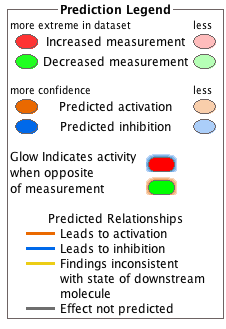


**Supplementary Figure 5b**

**b.** IPA mechanistic network depicting the interaction between upstream regulators that explain the observed changes in the WT specific gene list: genes significantly differentially expressed (adjusted p<0.05) with a |FC| > 2.0 uniquely in WT, E16.5 vs. E14.5. Regulators that are involved in the “cell death” functional category are highlighted (in purple border). A solid line implies a direct interaction while a dashed line implies an indirect interaction


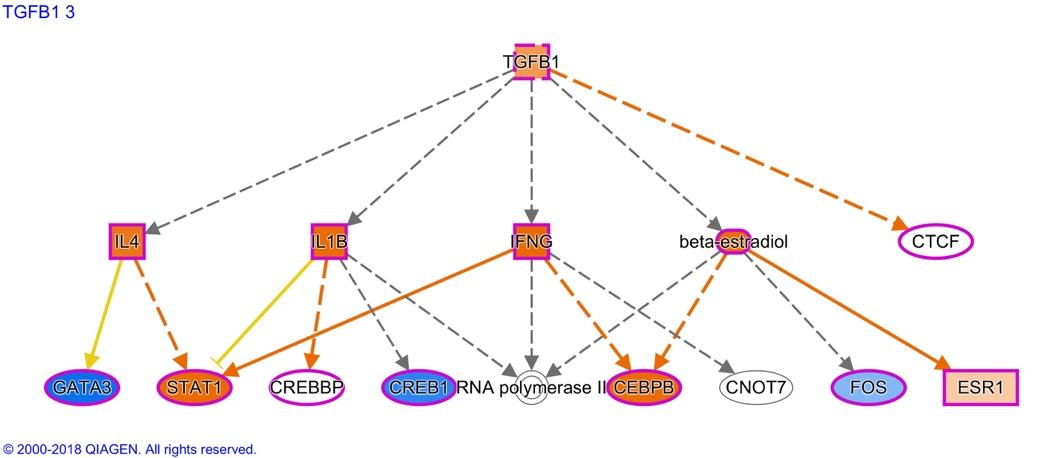

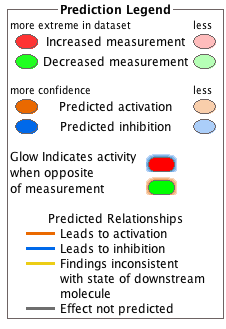


**Supplementary Figure 6.** Target molecules (upstream analysis) of TGF-β3 signaling pathways in WT palates that are up (pink) /down (green) regulated in response to TGF-β3. A solid line implies a direct interaction while a dashed line implies an indirect interaction

**
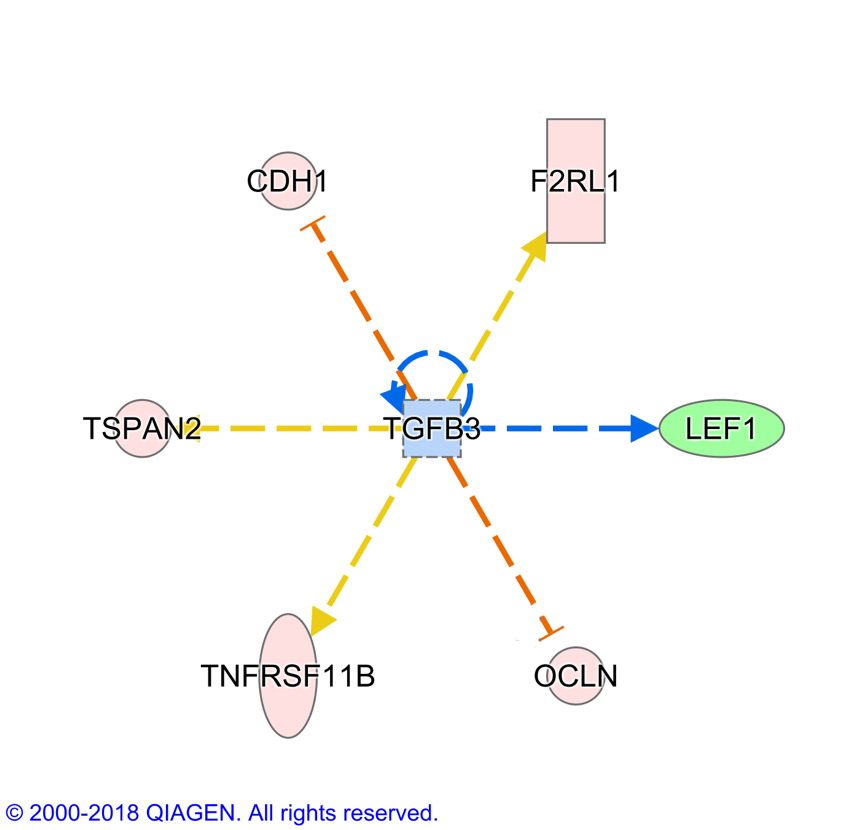

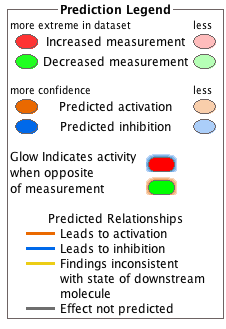
**

**Supplementary Figure 7.** IPA Interaction Network Analysis for the HM specific gene list: genes significantly differentially expressed (adjusted p<0.05) with a |FC| > 2.0 uniquely in HM, E16.5 vs. E14.5. Shades of red implies upregulation and shades of green implies downregulation. Genes that are also involved in major non-Smad pathways (ERK/MAPK, p38MAPK and PI3-AKT) in palatogenesis are indicated with links to the corresponding pathways. The p38MAPK gene does not appear in the network. A solid line implies a direct interaction while a dashed line implies an indirect interaction


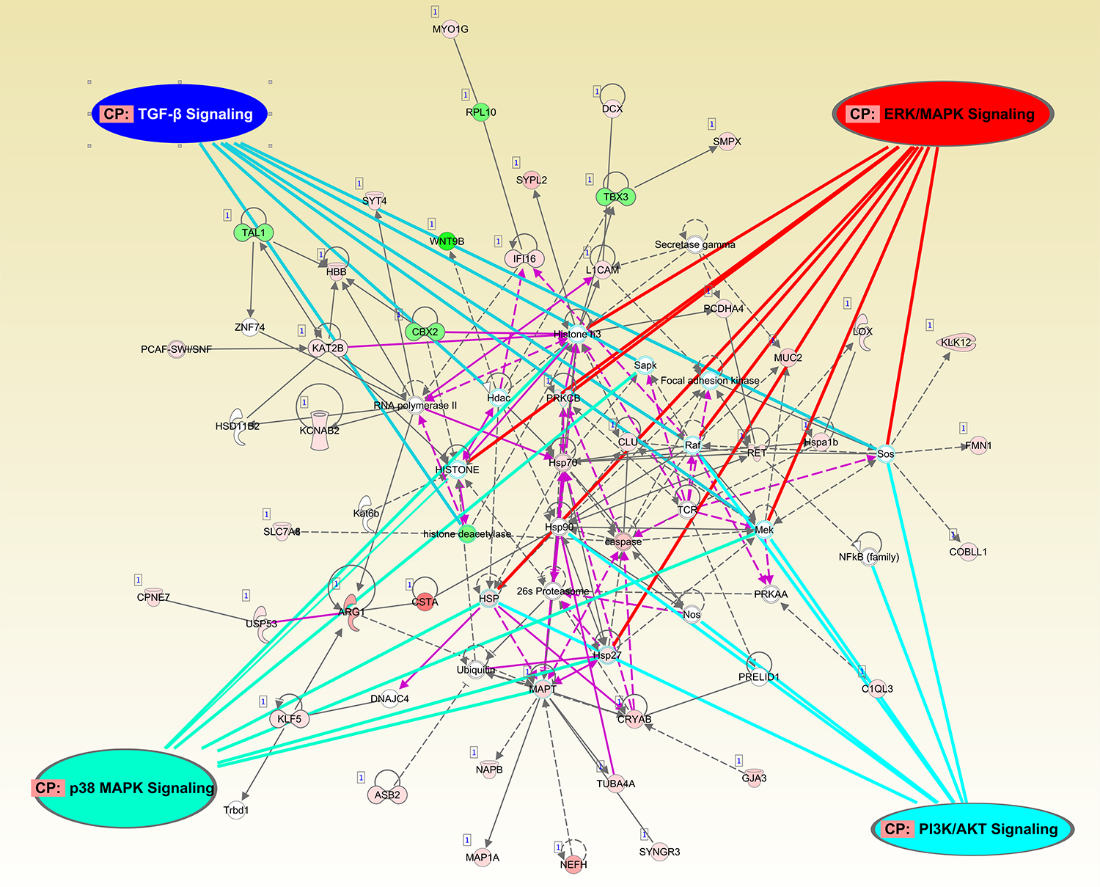


**Supplementary data: (attached excel file):**

A complete list of differential expression and analysis SDEGs, enriched GO categories and KEGG pathways.
